# How databases inform clinical management – insights from ozgrow

**DOI:** 10.1186/1687-9856-2015-S1-O4

**Published:** 2015-04-28

**Authors:** Ian Hughes, Andrew Cotterill, Cathy Choong, Paul Hofman, Wayne Cutfield, George Werther, Maria Craig, Christopher Cowell, Mark Harris

**Affiliations:** 1 OZGROW & Mater Research, UQ Institute, Brisbane, QLD, Australia; 2 Mater Children’s Hospital, Brisbane, QLD, Australia; 3 Princess Margaret Hospital Perth, Perth, WA, Australia; 4 School of Pediatrics and Child Health, University of Western Australia, Perth, WA, Australia; 5 Liggins Institute, Auckland, New Zealand; 6 University of Auckland, Auckland, New Zealand; 7 The Royal Children’s Hospital, Melbourne, VIC, Australia; 8 Murdoch Children’s Research Institute, Melbourne, VIC, Australia; 9 Institute of Endocrinology and Diabetes, The Children’s Hospital at Westmead, Sydney, NSW, Australia; 10 School of Women's and Children's Health,, The University of Sydney Medicine, Sydney, NSW, Australia; 11 Institute of Endocrinology and Diabetes, The Children’s Hospital Westmead, Sydney, NSW, Australia; 12 Discipline of Paediatrics and Child Health, University of Sydney, Sydney, NSW, Australia; 13 Lady Cilento Children’s Hospital, Brisbane, QLD, Australia

## 

Recombinant human growth hormone (GH) has been used as an effective treatment for short stature since the late 1980’s. Although clinical trials were crucial in developing GH treatment strategies, database analyses have the potential to provide additional information with regard to treatment efficacy and safety. The OZGROW database was established in 1989 as an initiative of the Australasian Paediatric Endocrine Group (APEG) to allow monitoring of children receiving GH under the national Pharmaceutical Benefits Scheme (PBS). There are currently (June 2014) 1948 children receiving subsidised GH treatment in Australia and the OZGROW database contains records from 6959 individuals. This unique resource has allowed a number of clinically relevant questions to be addressed.

Defining short stature and therefore eligibility for GH treatment relies on the use of appropriate growth standards. There has been debate as to whether the World Health Organisation (WHO) or the Center for Disease Control (CDC) growth charts best describe the heights of contemporary Australian children. There appears to have been a positive secular trend for height in Australian children such that both charts under-estimate the number of children eligible for growth hormone treatment.

Ascertainment bias has been proposed as an explanation for the fact that more boys than girls receive GH treatment. Analysis of the OZGROW database demonstrated that ascertainment bias did not appear to be a major factor explaining the gender discrepancy. It is possible that biological factors influencing the tempo of growth and development may contribute to the greater number of boys receiving growth hormone.

Another issue that has complicated comparisons of international studies of growth hormone response has been the different dosing schedules used. A simple method for converting weight and surface area based doses was therefore developed. Further analysis suggested that the surface area based protocol used in Australia may disadvantage older children, given that the dose per kilogram reduces over time even if the dose per meter squared remains constant.

The importance of commencing GH treatment early and maximising height gain during the first year of treatment was also highlighted by analysing GH responsiveness in a number of conditions including Turner syndrome, Prader-Willi syndrome and idiopathic short stature. Ensuring that referring physicians are able to diagnose these conditions in a timely fashion therefore remains a priority.

Although the OZGROW database has allowed clinically relevant questions to be studied there remain a number of challenges, particularly with regard to long term follow up.

